# Cancer of Liver and Pancreas, Large Collection of Calculi in Gall-Bladder†Read before Buffalo Medical Club, January 19, 1881.

**Published:** 1881-02

**Authors:** Thos. Lothrop


### CANCER OF LIVER AND PANCREAS, LARGE COLLECTION OF CALCULI IN GALL-BLADDER.†

REPORTED BY THOS. LOTHROP, M. D.

Mrs. K., aged 57, the mother of twelve children, who had been a vigorous and active woman, gave evidence of failing health for more than a year prior to the time she came under my observation. Without any obvious external cause, she suffered at times with great distress after eating, then with loss of appetite, diarrhœa alternating with constipation. With

† Read before Buffalo Medical Club, January 19, 1881.

these symptoms, the skin assumed an icteroid hue, and there was a general lassitude. A change of air was recommended and the patient passed the summer of 1879 in the country. Returning early in September, in a condition unimproved by the change, and with sufferings rather intensified, she sought medical advice, but without relief. Nov. 22, 1879, the patient was placed under my charge. The case presented the following symptoms: intense pain, at times paroxysmal, in left lumbar region, shooting down the left groin, nausea with frequent vomiting of ingesta, bowels constipated, circulation feeble, and general condition of the system unfavorable; the heart sounds, though feeble, were normal; there was a slight bronchitis with grayish-white, tenacious sputa; the urine was rather scanty, often of a dark color, due to bile-pigment; then again turbid, owing to the presence of the red-sediment of uric acid; the stools were hard and of a clayey color; no œdema of extremities, nor could fluid be detected in abdominal cavity. At this time no positive evidence of hepatic disease could be detected by examining the right hypochondriac region. The symptoms were entirely negative, with the exception of the severe pain in the left inguinal region which increased with the subsidence of her strength. Later, however, a distinct enlargement was felt below the margin of the right ribs, but without much pain on pressure. This was regarded as hepatic, and afforded an explanation, not before clear, of the icteroid hue of the skin, the clay colored stools and the bile-pigment in the urine.

The patient's condition became more and more unfavorable, and the prognosis, concurred in by Dr. Boardman, who was called in consultation, was grave. Medical treatment served only to alleviate her intense inguinal pain, without ameliorating the other negative symptoms or checking the progress of the case towards a fatal result. Death occurred Jan 12, 1880.

Dr. J. W. Keene, who at my request made the post-mortem examination, has kindly furnished the following report, which solves an interesting case, not possible to diagnose prior to death.

Jan. 13, 1880. Autopsy 3 P. M., twenty-four hours after death. Usual cadaveric rigidity; emaciation marked, but not extreme; appearance of skin as usual in persons of light complexion. On both legs, immediately above knee joint, were patches of red injected vessels of the arborescent form described as occurring after death from lightning. Nothing else remarkable externally.

On opening the abdomen, the liver was found slightly enlarged, solidified and friable, its peritoneal covering closely adherent and, when removed, showing a coarsely granular surface beneath. Numerous nodules, supposed to be cancerous, from the size of a walnut to that of the fist, were found pervading all parts of the viscus. The gall-bladder was enlarged to about twice its normal size, and projected considerably below the free anterior border of the liver. Its walls were opaque white and thickened in places by cancerous deposit to the feel of cartilage. The point projecting below the edge of liver was especially thick and cartilaginous, and was felt through the abdominal wall before death. The cyst was filled with a glairy, transparent substance, like the white of egg, and contained a large number of calculi, from the size of bird shot to that of a filbert. The hardened prominent point of the cyst contained a calculus almost completely encysted as large as a hickory nut; several others were found in pockets in the walls of the cyst. Total number of calculi found was 163, weighing 560 grains, Their composition was cholesterine. The cystic duct was impervious. The spleen was filled with similar deposits to those found in liver.

The pancreas was closely adherent to adjacent structures and of a firm, hard feel. On section, it was found to be made up of a material resembling brain in appearance, but of a much firmer consistency. The left kidney showed the presence of the same deposit, which pervades the other organs, but to a less degree. The largest nodule was about the size of an English walnut, and several smaller ones were observed. The right kidney showed very slight encroachment of the same disease. The pelvis and ureter of both were normal. Bladder and uterus exhibited nothing remarkable. Heart was normal. Lungs healthy, except that a few small shot-like nodules were found in them. Stomach, intestines and omentum were normal. None of the cancerous deposit was found elsewhere.

